# Unpacking the value of pharmacists in team-based primary care in British Columbia, Canada: a qualitative evaluation

**DOI:** 10.1186/s12875-025-03131-2

**Published:** 2025-12-09

**Authors:** Arwa Nemir, Anupama Salil, Anita I. Kapanen, Peter J. Zed

**Affiliations:** 1 Faculty of Pharmaceutical Sciences, University of British Columbia, Vancouver, BC Canada; 2 Department of Emergency Medicine, Faculty of Medicine, University of British Columbia, 2405 Wesbrook Mall, Vancouver, BC V6T 1Z3 Canada

**Keywords:** Primary care pharmacist, Pharmacy practice, Patient-centred care, Interprofessional teams, Comprehensive medication management, Team-based care

## Abstract

**Background:**

The pharmacy profession has been continuously evolving, redefining pharmacists’ scope of practice within various practice settings to meet the ongoing societal needs, demands and patient expectations. Pharmacists are increasingly becoming part of primary care teams in countries such as Canada, the United States, the United Kingdom, and Australia. In British Columbia, Canada, primary care clinical pharmacists (PCCPs) were incorporated into developing primary care networks (PCNs) over a three-year period. Our project aimed to gain insight into how the role of PCCPs is perceived by their interprofessional team (IPT) members in addition to how the pharmacists themselves view their contribution to team-based primary care.

**Methods:**

This project was conducted as part of the evaluation of the Pharmacists in PCN Program and was informed by Qualitative Description methodology. Data was collected at two time points during program implementation. Participants included program team members, PCCPs and PCN members (administrators, IPT members, and prescribers) who were invited via e-mail to participant in virtual individual interviews and focus groups.

**Results:**

Prescribers and PCN IPT members felt supported when collaborating with PCCPs for the shared care of mutual patients. Additionally, PCCPs believed that team members valued their role. There was a recognized need for PCCPs to be co-located in clinics as efficient, reliable, and accessible team members owing to their role in medication management, offering patient-centred recommendations and follow-up care plans to optimize medication regimens, providing patient education which empowers patients to advocate for their health needs, prioritizing patient autonomy, decreasing the amount of time prescribers and IPT members spend on complex patients, and supporting patients who do not have immediate access to a family physician.

**Conclusion:**

Perceptions of the PCCP’s contribution to IPTs were overwhelmingly positive. The PCCP’s specific role in addressing aspects of patient care that other health care providers may overlook, and their possession of knowledge, expertise, and skills which fall outside other team members’ scope of work has led to their recognition as an essential team member. However, more research is needed to look beyond the pharmacist’s impact on providing longitudinal medication management, as perceived by all members of the primary care team.

**Supplementary Information:**

The online version contains supplementary material available at 10.1186/s12875-025-03131-2.

## Background

Pharmacists are considered among the most accessible health care providers [1, 2]. Accordingly, the pharmacy profession has been continuously adapting, redefining pharmacists’ roles and scope of practice within various practice settings to meet the ongoing societal and patient demands and expectations [3]. Pharmacists are well-equipped with the knowledge, expertise, and skills and play a fundamental role in providing comprehensive medication management (CMM) which maximizes patient health outcomes by identifying and resolving drug therapy problems (DTPs) [4]. Comprehensive medication management is a structured patient-centred approach that ensures pharmacists evaluate a patient’s medications for proper use, effectiveness, safety, and potential barriers to adherence [5, 6].

Extensive research supports the benefits of including pharmacists in interprofessional primary care teams to complement the role and skillset of other team members, specifically physicians and nurse practitioners [7]. In primary care settings, pharmacists are actively involved in enhancing care for patients with chronic conditions, promoting health and wellness, and contributing positively to quality measures by ensuring safe and effective use of medications [7]. Consequently, pharmacists are increasingly becoming part of primary care teams in countries such as Canada, the United States (US), the United Kingdom, Australia and China [8–11].

In Canada, both family physicians and pharmacists have supported the incorporation of pharmacists into interprofessional teams (IPTs) across the country [12–14]. The extent of pharmacist integration varies by province, including Ontario, Quebec, Alberta, and British Columbia (BC) [15]. In the province of BC, the health care vision emphasizes collaboration among diverse health care providers, ensuring they work together and complement one another within family medical practice settings—called Patient Medical Homes (PMHs)—and across defined geographic areas known as Primary Care Networks (PCNs) [15]. A PCN is a service model that links and integrates patient care within a particular geographic region, bringing together PMHs, regional services such as hospitals, specialized community programs, and home and community care, as well as the broader health care system [16].

From October 1, 2020 to September 30, 2023, the Faculty of Pharmaceutical Sciences at the University of British Columbia (UBC) led the implementation of the Pharmacists in PCN Program (the program), a three-year initiative that also included a comprehensive evaluation [17]. Funded by the BC Ministry of Health, the program integrated Primary Care Clinical Pharmacists (PCCPs) into 47 PCNs across BC, where 59 PCCPs delivered more than 24,000 patient appointments [18, 19] and identified over 30,000 DTPs requiring intervention, monitoring, or education [19]. Developed as part of the province’s broader strategy to strengthen patient-centred team-based primary care, the program marked the first full integration of such teams within PCNs [16]. Implementation was a collaborative effort involving UBC, the BC Ministry of Health, Divisions of Family Practice, regional health authorities, and PCNs to embed PCCPs as core members of the IPT.

The objective of this evaluation was to gain an understanding of how the value and role of PCCPs in team-based primary care is perceived by diverse program stakeholders and team members, including how PCCPs themselves view their contribution to interprofessional primary care teams.

## Methods

### Study setting

We report findings of the evaluation of the Pharmacists in PCN Program at two time points, T1 (June – July 2022) and T2 (June – October 2023) on the role PCCPs played within the program as perceived by PCCPs themselves, UBC team members and PCN members including administrators (managers, directors, leads, and coaches), IPT members (social workers, respiratory therapists, physical therapists, occupational therapists, registered nurses, counsellors, and dietitians) and prescribers (family physicians and nurse practitioners). In T1, we captured early stakeholder experiences and perspectives over the first 18–20 months of program implementation and in T2, we explored the final 16–18 months as well as terminal experiences at the end of the three-year implementation period.

### Participant recruitment

All UBC program team members who were actively involved in program implementation and PCCP integration were deemed eligible. PCCPs were eligible to participate in a focus group interview if they have been working with their PCN for at least six months. All PCN administrators and IPT members from every PCN community were eligible if they were identified by the program team and PCCPs, respectively, as having interacted and are familiar with the role of the program and PCCP. Eligible participants from both groups were first asked to participate in a survey [20] then a portion of survey respondents provided consent to be contacted for an individual virtual interview. Moreover, prescribers were categorized according to their level of interaction with the PCCP/referral volumes (low – high), with random selection of eligible participants among the upper-limit of high-medium volume categories.

### Data collection

A purposive sample of each participant group was recruited. Participants received an e-mail invite to a virtual interview over Zoom. All focus groups and individual interviews were conducted by two researchers (AN/AS). The sessions were facilitated using interview guides developed by the evaluation team. The interview guides were informed by the evaluation framework, literature review, and team discussions and differed depending on the participant group (see Supplementary Material). All interviews were audio-recorded and transcribed verbatim by a third-party professional transcription company. In order to protect participant privacy, all participant names and other identifying information were eliminated from all transcripts, and the transcribing company was not granted access to any of the participants’ personal data or videos.

### Data analysis

Analysis of interview data was qualitative and iterative, taking place concurrently with the interviews. Our analysis employed content and thematic analysis and was guided by the Qualitative Description (QD) methodology [21, 22]. We used content and thematic analysis as complementary methods to align with QD methodology and support the objectives of our evaluation and level of interpretation desired [23]. Content analysis was applied to categorize patterns and describe the presence, meanings, and relationships of words and concepts within and across diverse participant datasets [23, 24]. Thematic analysis allowed for a deeper level of interpretation, focusing on the identification, analysis, and reporting of themes while providing a rich and nuanced understanding of participants’ perspectives and experiences through inductive development and refinement of themes [25, 26].

Both AN and AS performed the data analysis, starting by familiarizing themselves with the data through being immersed in all interview transcripts to gain an overall sense of participants’ accounts. Written reflections from interviews were also reviewed. AN and AS met to initially code a subset of transcripts line-by-line to capture meaningful segments of text and inductively develop an initial coding framework which guided the entire data analysis.The coding framework was incorporated into the NVivo 12^™^ program. Codes that were conceptually similar were grouped together to create categories. Categories were then organized into preliminary themes through identifying patterns in data within the same participant groups or across different groups. Themes were developed and refined in relation to the coded data and the full dataset to ensure they accurately represented commonalities and differences of perspectives and experiences from the diversity of participant groups interviewed. Longitudinal comparisons were also made to identify trajectories or shifts in participants’ perspectives and/experiences over time for those participants who were interviewed at both time points. Final themes were defined, named, and clearly described. Exemplar quotes were added to complement each theme with some quotes paraphrased in-text rather than presented as they are for coherence and clarity. Credibility was enhanced through having AN and AS independently code the transcripts, practice reflexive journaling, and have regular meetings which allowed the revision and refinement of initial codes and coding framework. Open communication and debates between AN and AS were used to reach consensus and settle any disagreements or uncertainties in coding.

### Ethical considerations

The study sponsor (BC Ministry of Health) completed a privacy impact assessment and the University of British Columbia Behavioural Research Ethics Board determined that no ethical approval was needed for this part of the evaluation based on Article 2.5 of the Canadian Tri-Council Policy Statement: Ethical Conduct for Research Involving Humans (TCPS2).

## Findings

In T1, we conducted individual interviews with six UBC program team members and two focus group interviews with fifteen PCCPs working in PCNs across three BC health authorities. In T2, we conducted individual interviews with four UBC program team members, one UBC program team focus group and four focus group interviews with thirty-nine PCCPs representing all five BC health authorities. In addition, we interviewed thirteen PCN administrators, twelve PCN IPT members, and eleven prescribers. Six main themes were inductively derived regarding the perceived benefits and contribution of PCCPs to team-based primary care, and included; (i) PCCPs Recognized for Pharmacy-Related Knowledge, Skills and Specialized Training; (ii) PCCPs Providing Holistic and Comprehensive Patient-Centered Care; (iii) PCCPs as Reliable Team Players; (iv) PCCPs Empowering Patients to Advocate for Their Health Needs; (v) PCCP's Role in Caring for Unattached Patients; and (vi) PCCPs Optimizing the Patient Care Process. Overall, these overarching themes highlight the pharmacist’s perceived role in primary care as a scholar, patient-centred health care professional,collaborator, supporting and promoting patient autonomy, bridging the gap in health care access and longitudinal care, and being an efficient health care professional, as viewed by PCCPs themselves and program stakeholders.

### Theme #1 - PCCPs recognized for pharmacy-related knowledge, skills, and specialized training

Overall, PCCPs were highly regarded as an integral part of the IPT owing to their expertise, clinical and non-clinical skills, knowledge of CMM, and specialized training, especially when caring for complex patients on polypharmacy. Within the IPT, PCCPs were recognized by other health care providers for those exceptional care qualities that are specific to their training and extensive pharmacotherapy knowledge base.

*“[Pharmacists] have this unique knowledge base so pertinent to patient care. We can only do so much without them. Social workers*,* nurses*,* pharmacists*,* they have to be at the table. We cannot function without them because that piece is so part of patient care. It is just critical. Doctors are not that reachable. You cannot just call them up. But with the pharmacist you can have a different type of conversation. There [are] so many people out there on the wrong medication*,* on too many medications*,* and they are suffering. They are not being treated properly because doctors are busy*,* they do not have the time to sit around and review everybody’s medications and come up with a plan that would work better so*,* having a pharmacist to really take the time to listen to what I have to say and then talk to the patients*,* listen to what they have to say and come up with a plan to at the end of the day make their lives better.”*[PCN IPT Member #2].

*“Often it just seems like when people have multiple specialists*,* they will just take on medication after medication and there is not really somebody that is zoomed out and looking at what are all these meds across all these different specialties and family practice. Pharmacists can come in laser-focused*,* and they can see all the pieces to these puzzles that get missed.”* [PCN IPT Member #11].

The PCCP’s up-to-date knowledge and expertise about medication adverse effects, drug interactions, drug dosing and administration, treatment options, and medication pricing and coverage were also deemed valuable by all PCN member groups since it was considered as knowledge that neither prescribers nor IPT members possess.

*“I think a lot of times with the chronic disease nurses*,* they ask us about* *how they get their medications*,* how things are covered*,* things like that so they can make the best recommendations.”* [PCCP #5 in Focus Group #2].

*“They have knowledge and expertise that only helps us support the clients better. I have the experience with the disorders recognizing the symptoms. I know the treatments that were available*,* the common ones*,* but there are always new treatments available. They would know whether this treatment might be suitable or if they are on this*,* this does not work so they know about  the drug interactions*,* they know about the new medications coming out*,* so they are up on all the latest information.”* [PCN IPT Member #12].

*“The physicians are overwhelmed*,* patients are a lot more complex*,* and polypharmacy is huge. A pharmacist is adding great value and physicians do not have enough time to spend with a patient to educate them on all the medication changes*,* all the interactions. This pharmacist role plays a huge part also with getting the patient more comfortable with their changes in meds and their health and diseases. It is a valued team-based care member.”* [PCN Administrator #5].

In particular, PCCPs were recognized for aspects of patient care that pertains to their pharmacist role such as extensive knowledge on medications and specific disease conditions, deprescribing, CMM, and medication reconciliation.

*“It’s really helped my management in terms of pharmacologic recommendations and I really rely on that consultation to be able to make some challenging decisions with whether it is deprescribing or prescribing a medication that I am not entirely familiar with. I am usually able to call the patient that same morning or afternoon and implement recommendations. It certainly does cut down on my time with patients if there is follow-up needed. Being team-based care there is as primary care practitioners*,* we are not experts in every single area of pharmacological management.”* [Prescriber #11].

*“It is incredibly valuable just to have somebody with all of that knowledge dedicated to that one topic. It is so specialized but so integral to the whole picture. It is been amazing I mean just to be able to have somebody to take the time to do the lists*,* to look at the reconciliation*,* to ask the questions of the patients about other things that they consume or you know supplements or just the full picture that often just primary care providers do not have the depth of knowledge and do not have the time.”* [PCN Administrator #13].

Furthermore, PCCPs were commended for their role in providing patient education, and improving patient care while supporting prescribers, especially new and retiring physicians, and nurse practitioners and utilizing specialized knowledge that is unique to the pharmacist’s training and expertise.

*“Oh*,* it is amazing*,* there is so much value. I mean for our physicians too. Some of them [physicians] have worked with dispensing pharmacists where they could call and ask questions but having this new resource has been really amazing. For our new physicians that are starting practice*,* it can be quite overwhelming. Having the additional support of having someone look at the medication list has been seen as an incredible value.”* [PCN Administrator #6].

*“Especially when it comes to education because there is not a very reliable way for patients to access that kind of information about psychiatric medication. For example*,* waiting six weeks for the full effect to [take] place*,* and then experiencing frustration beforehand. Some clients are concerned about addictive properties of these medications that are really within a pharmacist scope*,* being able to provide education to the clients that a lot of us other clinicians just simply cannot provide. It is out of our scope. Being able to offer medication reviews for our clients. Being able to be an additional support or advocate and someone who can participate in case consults with the doctors in order to ensure that the clients are getting the best care.”* [PCN IPT Member #1].

The PCCP’s medication expertise was particularly viewed as a contribution to providing quality patient care, especially in managing complex polypharmacy, in addition to their role in optimizing patient care through conducting medication reviews, deprescribing, and supporting patients in achieving their personalized health goals.

*“It is a really important role to the clinical team*,* particularly in the setting of complex patients within particular polypharmacy. Looking at med management and a lot of our patients have low literacy. To even look at supporting them with med management*,* even when we are blister packing medications*,* looking at deprescribing*,* looking at just an overall review of multiple medications if there is something that we could be tweaking. If there is folks who are not meeting their targets in terms of A1C or other targets*,* what can we be doing to support. I think there is absolutely a role.”* [Prescriber #5].

PCCPs also supported prescribers and IPT members in caring for patients with specific disease conditions owing to having additional certifications (e.g., diabetes educator) and specialized training.

*“Our clinical pharmacist has got herself certified in diabetes management as well. She has been instrumental in getting us started on things like [medication name] when it was brand new to us a year ago. Changing drugs*,* looking at interactions between drugs. I want to start this*,* but I know it is going to interact with that*,* what we do? And she will come back with suggestions.”* [Prescriber #1].

*“The PCCP has her certified diabetes educator (CDE). She can do the diabetes medication adjustments for people. That is really helpful especially in this area because it is rural so we do have a diabetes education clinic where people would be referred to see a nurse diabetes educator and a dietician*,* but they have really limited capacity to see people. Having the PCCP who can talk to them about the medication knowledge in that area is really helpful so that I can still work with them on the food part without having to feel like I am working out of my scope and trying to give them medication recommendations*,* which I wouldn’t do*,* but just trying to manage their intake and the insulin can sometimes be challenging.”*[PCN IPT Member #4].

From the perspectives of PCCPs and how their role has been perceived by their colleagues, PCCPs described that PCN IPT members and prescribers were comfortable approaching them and particularly appreciated their medication-related knowledge.

*“I feel valued. I know they come to us with questions fairly regularly and I feel like they are comfortable asking us questions candidly in a hallway hoping for an answer pretty quick. I think the other members of the team are uncomfortable in the realm of medications. I have heard them mention, "why cannot you prescribe? Why do you know all this and why do you have all this information? You are giving this recommendation; why cannot you just put it to fruition?" I avoid that conversation because it is a long one*,* but I hear it being mentioned.”* [PCCP #1 in Focus Group #1].

*“Where I am, we are highly regarded. We work in a hub so we are all in the same place so a lot of the other clinicians would just come into our office and then chat with us about drug-related things*,* ask us pharmacy questions. When I am co-located*,* the nurse practitioners especially ask a lot of questions. I am really happy to walk them through different treatment algorithms and things like that.”* [PCCP #5 in Focus Group #2].

According to PCCPs, PCN IPT members' appreciation of PCCPs was reflected through increase in referrals and seeking the PCCP's help with medication-related questions.

*“I would say out of the PCNs we receive the highest volume of referrals from the mental health nurse*,* from the physiotherapist*,* just anytime there is medication involved. I almost feel like they view us like a sub-prescriber. They cannot reach directly to the doctor so they’ will ask us a question. I do not know if that is a healthy mentality but it does happen so we end up answering a lot of drug related questions and that in turn does generate referrals so it is kind of a win-win.”* [PCCP #3 in Focus Group #1].

In addition, some PCCPs felt valued by community pharmacists owing to their knowledge, access to information resources which community pharmacists do not always have, connection with prescribers, dedicated time to patient care, and role in medication reviews.

*“I felt that we are valued*,* especially in terms of the clinical background*,* but also having basically access some of the patient resources that the community colleagues do not have and also the time that we have or the better connection from the referring physician. I feel the community pharmacist understand  that it is much easier for the PCCPs to be involved in medication reviews. Usually*,* the community pharmacists are extremely busy and they really cannot afford spending too much time on certain issues.”* [PCCP #6 in Focus Group #2].

### Theme #2 - PCCPs providing holistic and comprehensive patient-centered care

PCCP appointments were structured to provide holistic care, often for elderly patients with multiple co-morbidities and are taking many medications. Unlike in a community pharmacy, appointments with a PCCP were longer (30–60 min) so they have more capacity within their appointment structure which allowed for a comprehensive medication review. PCCPs were also found to utilize their skills to their full potential and scope of work to provide longitudinal patient-centered care based on evidence while being part of the IPT.

*“It is not always necessarily the medical complexity of the patient but just the time intensiveness of what that task that we are being referred is going to take just because we have that opportunity to sit down with that person for a longer time than their provider does so*,* we provide more comprehensive education or plan building or whatever it may be*,* even if the condition itself is not that complex. I have seen quite a few people for weight management obesity stuff and the medications are maybe not complex in that situation but taking the time to kind of go through that comprehensive education process with the patient.”* [PCCP #2 in Focus Group #2].

*“Quality of care comes with evidence-based*,* patient-centred and I will see that in the notes too. For example*,* [the PCCP] may really feel like someone needs to be on a statin and will educate the patient why [they] think that*,* but they might say I actually do not want to be on a statin and so that is patient-centred care to honor the wishes and I can see that reflected in [their] plans. Then the follow up is a big piece. They just do not feel like it is a one and done. Everyone has skills to contribute*,* everyone should play and should utilize their skills to their full potential*,* their full scope.”* [Prescriber #3].

*“I see primary care pharmacy practice as team-based primary care as the truest definition of what primary care should be. I see that as providing comprehensive medication management to patients alongside a care team and over time. I think longitudinally and having that relationship is a big piece of it and that of course applies in my mind to a primary care setting. Our patients are accessing care from a family physician clinic*,* nurse practitioner clinic*,* primary health care clinic*,* not in a hospital specialty setting or an ambulatory care setting*,* but a place where they come*,* they access care and they have access to a full team and that is an ongoing relationship not a one-off encounter.”* [UBC Team Member #5].

In comparison with family physicians, PCCP’s accessibility and scope of work allowed for a more optimized and personalized health care especially in conducting medication reviews, deprescribing, and creating individualized care plans. Some PCN members also valued the PCCP’s humanistic approach to care which was reflected in fostering a PCCP-patient relationship built on respect, kindness and patience. Specifically, PCCPs were commended for providing care that is tailored to patient-specific factors, values, needs, and preferences while respectfully and actively listening to the patient perspectives and including them in the conversation.

*“Because they look at the client not only specifically one symptom*,* it is a more broad review of everything. That is what we do*,* we do holistic assessment in their homes so we present this client as a whole*,* whatever is home environment*,* whatever is family support*,* whatever is the symptoms they experience*,* what happened in the past*,* how it affects them in the present and they take it into consideration. I really like the pharmacist’s recommendation*,* it is so long*,* especially [PCCP Name]*,* it is unbelievable. I learn so much to be honest because they address every symptom from different angles.”* [PCN IPT Member #7].

*“Best treatment in terms of drug choice*,* and this is not always academic best treatment. It is also what is best for the patient. What is practical for them*,* what they tolerate*,* what they do not tolerate. Why is this person on a long-acting beta-agonist and a long-acting muscarinic agonist? Why aren’t we on a combination inhaler? Those kinds of things were very helpful. I have got a fairly psychiatric-heavy practice and side effects from things the atypical anti-psychotics and changing from one to the other without coming totally off and risking everybody falling out of the wagon during a med change.”* [Prescriber #1].

### Theme #3 - PCCPs as reliable team players

PCCPs were recognized as team players by PCN administrators, IPT members, and prescribers, supporting their colleagues in their roles and collaborating with them for the shared care of mutual patients during referrals and discharge. Specific pharmacy-related skills which PCCPs were valued for by their teammates included professionalism and efficient and organized oral and written communication.

*“Keeping things like communications*,* tidy and transparent and everybody knows where that patient is in whatever aspect is huge. Being able to communicate directly with the PCCP on findings and recommendations and med lists now that are totally accurate instead of ‘we are not sure*,*’ super valuable.”* [PCN Administrator #13].

*“With patients*,* when I tell them I recommend a referral to the pharmacist*,* I think for knowing that we are working as a team and that we trust each other*,* that we are all part of this team that is working together for their good to better their lives. It just adds to my practice. I do not feel alone*,* I do not feel like I have to do everything. I know I have people I can consult with and even if [PCCP Name] does not get involved*,* I can still call her and ask her questions and consult with her and get information quickly*,* easily.”* [PCN IPT Member #2].

*“They are so well appreciated and their role is so important and the work has resonated very strongly with the providers. They are working well with the team*,* everybody loves them*,* they love the work they do*,* they recognize how important the work they do so it has just been really great.”* [PCN Administrator #10].

PCCPs were also viewed as being approachable and open to discussions with their teammates in addition to being accessible when needed, providing timely support to their teammates by promptly responding to questions and addressing referrals from IPT members.

*“I cannot imagine running our primary care network without them [PCCPs] honestly*,* I rave about their professionalism all the time and when I am speaking to other primary care networks that are starting to develop their program*,* one of my first things I say to them is, "you need to get a clinical pharmacist." I say that they’ are next level*,* they’ are always on board. I have had three different clinical pharmacists that I have worked with here and all of them are just so intelligent and so good at their job*,* always willing to jump in and help.”* [PCN Administrator #12].

*“I am just boggled by what [the PCCP] can just pull out of the back of his brain. I find*,* knowing that I have that personal relationship with somebody*,* it is different than just cold calling a pharmacy. I feel like I can ask stupid questions because [they are] so personable and I have asked stupid questions and acknowledged it and  [they are] graceful and respectful and I like the personal relationship part.”* [PCN IPT Member #5].

*“I have worked with two different PCCPs*,* one in each area. Both of them I have found to be very thorough, easy to work with and helpful and open to answering questions. Notes that I have seen from people that they have referred to me or that we have both been seeing are always very thorough.”* [PCN IPT Member #4].

### Theme #4 - PCCPs empowering patients to advocate for their health needs

PCCPs supported and promoted patient autonomy to make informed decisions about their health through having conversations with them regarding their medications and addressing their concerns. Consequently, patients were empowered to educate their IPT members to help with their care owing to the PCCP’s involvement in delivering patient education, both pharmacological and non-pharmacological.

*“[Clients] may even reflect back to me things that they heard from the PCCP that they internalize pieces of education that they got or information that they are going to carry forward. We want to also be aware of that*,* so it sounds like little pieces of information*,* knowledge*,* and wisdom that the clients carry forward in their sessions it also makes my job a bit easier too because the PCCPs are helping in a therapeutic perspective as well and supporting the work that I do.”* [PCN IPT Member #1].

### Theme #5 – PCCP’s role in caring for unattached patients

In a system where PCCP adoption is fully acknowledged and appreciated, PCCPs are ideally positioned to steward unattached patients into continuous primary care in PCNs, in part by reminding physicians about how their knowledge and expertise adds value to patient care. Therefore, PCCPs play a key role in providing timely care and caring for patients who do not have immediate access to a prescriber. PCCPs shared that, through being able to practice to their full scope, their role contributes to the overall goal of the program which is optimizing patient-centred care in primary care settings while alleviating the burden on prescribers and allowing them to focus their efforts on tasks that are more specific to their scope of practice.

*“What was supposed to be happening is that based on the research and the evidence how many unattached patients there are by PCN. It’s a big problem. The idea of doing this team-based care is to reduce that number. The PCCPs are to help break down the barriers that we have had in this province*,* to help [family physicians] understand the value of a pharmacist regardless of which pharmacist it is. Help those  [family physicians] understand what a pharmacist can do.”* [UBC Team Member #4].

*“The patient receives much faster response to their concerns*,* to their health care needs. Unfortunately*,* waiting lists to see geriatric physician is really long*,* it is over a year and if the patient is waiting in pain for all this year*,* it is not good because they will go down really fast*,* they will have lower mobility*,* they will have much higher depression which may lead to some more confusion*,* etcetera*,* and high mortality. With the pharmacist readily available and more than willing to address the current situation by contacting family physicians*,* making suggestions. I think the patients are much better supported and overall*,* the seniors population in [PCN Name] benefits a lot.”* [PCN IPT Member #7].

The burden of unattached patients and connecting with prescribers who are not their long-term primary care providers (PCPs) is currently placed on community pharmacists. Consequently, PCCPs served as the bridge between physicians and community pharmacists thus ensuring continuous and safe care of complex patients.

*“All the faxes that [community pharmacies] are dealing with*,* communicating with prescribers could be reduced because we have upstream people working right there before the orders even go out*,* things will be done right. From a community pharmacy perspective*,* the dream is reducing our faxes*,* reduce our communications with prescribers*,* chasing prescribers*,* because you guys will get it right up front. The other things is*,* we got [doctors] that are not very cooperative but if you guys can get them cooperative then that relationship building and collaboration*,* that will be so good for the profession. In hospital*,* the [doctors] are used to working with the pharmacists*,* they depend on them*,* they expect them to be there. When it comes to in the community*,* not at all.”* [UBC Team Member #4].

### Theme #6 - PCCPs optimizing the patient care process

There were specific activities uniquely performed by the PCCP which some prescribers and IPT members considered to increase the efficiency of patient care, their satisfaction, and support the appropriate use of medications. These activities included following up with patients on new medications, monitoring for efficacy and safety, and providing patient education which is tailored to individual patients’ needs. PCCPs also optimized the efficiency of the patient care process by decreasing the amount of time their teammates spent on complex patients or those with specific health care preferences and/or needs.

*“I believe I had them follow up with some starting new medications that maybe are more prone to side effects. Have the pharmacist follow-up with people if they are having issues with side effects and what do they do. There is opportunity for group education*,* I can think there are some medications that regular follow-up would be helpful*,* saving time. Increasing appropriate use of medications*,* reducing unnecessary prescriptions and deprescribing.”* [Prescriber #2].

Furthermore, PCCPs played a role in having one-on-one conversations with patients about their medications which is time-consuming for IPT members and prescribers and falls outside their scope/expertise. IPT members believed that the PCCP’s contribution to the patient care process, especially by being medication therapy experts, helped alleviate the burden on busy prescribers by dedicating time to specifically address the patient’s medication-related questions and concerns.

*“Sometimes it is just nice for patients to hear it from somebody else and someone who just comes from a different perspective. They [family physicians] just do not have the luxury or time to sit down and really of sift through things. The fact that [the PCCP] has the in-depth knowledge and he is very up to speed too on research.”* [Prescriber #3].

*“Because doctors do not have the time or maybe even the knowledge to explain medications to people in their ten minutes they have with someone. People go away and they have no idea what it is they are taking or what it is for and a lot of times people stop taking it or titrate the dose and they do all kinds of stuff*,* right. So*,* it is essential.”* [PCN IPT Member #2].

## Discussion

Our findings demonstrate that all PCN members (administrators, IPT members, and prescribers) welcomed the PCCP as a member of the IPT and appreciated their role and value in optimizing the patient care process, improving quality of care, and providing individualized and comprehensive care. PCCPs were specifically recognized for several patient care-related activities which included, performing comprehensive medication reviews and medication reconciliation, offering patient-centred recommendations, providing patient education which consequently empowered patients to advocate for their health needs, supporting the efficiency of the patient care process by decreasing the amount of time the prescribers and IPT members spent on complex patients or those with specific health care preferences and/or needs, actively involving patients in discussions about their health and medications and addressing their questions and/or concerns, which is otherwise time-consuming for prescribers and IPT members, and/or is outside of their scope/expertise,supporting patients who do not have an immediate access to a prescriber, and demonstrating a team-based approach/philosophy. In addition, PCN IPT members and prescribers felt supported within their role when collaborating with PCCPs for the shared care of mutual patients and viewed them as reliable colleagues who optimized the efficiency of the patient care process. They also affirmed a recognized need for PCCPs in primary care settings owing to their unique knowledge, skills, and medication therapy expertise, which complements the role and skills of their teammates.

Our current work builds on evidence from other studies that explored primary care providers’ (PCPs) perceptions of the role and contribution of pharmacists in team-based primary care. Abbinanti et al. interviewed twenty-five PCPs working collaboratively with pharmacists in nine US-based university health clinics [27]. Participants highly valued the integration of pharmacists within their clinics, citing substantial benefits to provider efficiency, job satisfaction, and patient care while enhancing the overall effectiveness of primary care delivery[27]. Furthermore, participants consistently underscored the vital role pharmacists play in managing complex and chronic conditions, providing evidence-based care, and improving patient outcomes, highlighting how the presence of pharmacists on their team was also linked to reduced workload and burnout in addition to improved interprofessional collaboration [27].

Findings from our project also contribute to the growing international evidence-base supporting the integration of pharmacists into primary care teams, specifically navigating PCPs’ experiences with CMM services offered by pharmacists. In alignment with our work, PCPs considered pharmacists who provide CMM as accessible and collaborative team players complementing the PCP’s skillset owing to the pharmacist’s knowledge base on medications [28]. PCPs also valued the pharmacist’s role in enriching the physician’s knowledge [29–31] and confidence in prescribing [32], enhancing the treatment of complex patients [31], saving the provider’s time and alleviating their workload [33, 34] in addition to augmenting the physician’s ability to optimize patient care [29, 30]. However, future research should explore the pharmacist’s role in delivering CMM from the perspectives of both physician and non-physician members of the primary care team. This is important because existing evidence on the perceived benefits of pharmacists in team-based primary care largely focuses on physician-pharmacist collaboration, with limited attention to views of team members belonging to other healthcare professions.

### Strengths and limitations

To our knowledge, this is the most extensive qualitative work offering a thorough evaluation of the benefits of PCCPs and navigating numerous aspects of the pharmacist's contribution to team-based primary care as perceived by diverse participant groups of IPT members, including pharmacists themselves. Utilizing qualitative interviews and thematic analysis provided in-depth insights into participants’ perspectives. Furthermore, inclusion of multiple participant groups with different roles and responsibilities within the program and from various PCN locations ensured we captured a diversity in viewpoints and experiences to enhance the transferability and credibility of our findings and reduce sampling bias. In addition, collecting data at two time points captured changes in stakeholder perspectives over the course of program implementation.

This evaluation also has certain limitations worth noting. One is the potential for researcher bias, as one of the data analysts is a pharmacist whose professional background and prior views on pharmacy practice in primary care may have shaped the analysis and interpretation of findings. Although our findings may be relevant to other primary care teams—both within Canada and internationally—particularly those with comparable environments, infrastructure, and resources, they only reflect the first three years of program implementation and may not capture or convey longer-term experiences or perspectives. Another element that is specific to the evaluation we conducted is that, at the time of this project, the BC team-based care model was still developing, and certain PCNs might have been further along in their implementation than others. Furthermore, the sequence of IPT members’ integration was not consistent, so viewpoints of the temporal relationships with the maturation of PCN development and team construction might have impacted experiences and therefore warrant additional research.

## Conclusion

Perceptions of the PCCP’s contribution to IPTs were overwhelmingly positive. The PCCP’s specific role in addressing aspects of patient care that other health care providers may overlook and their possession of knowledge, expertise, and skills which fall outside their teammates’ scope of work has led to the PCCP’s recognition as being fundamental to primary care IPTs. Understanding the various ways primary care pharmacists contribute to the patient care process is essential to optimizing interprofessional collaboration in primary care settings.However, more research is needed to look beyond the pharmacist’s impact on providing CMM, as perceived not only by physician but also non-physician members of the primary care team.

## Supplementary Information

Supplementary Material 1.

## Data Availability

Data generated during and/or analysed during the current study are not available. However, inquiries regarding data can be directed to the corresponding author.

## Abbreviations

BC: British Columbia
CMM: Comprehensive Medication Management
DTP: Drug Therapy Problem
IPT: Interprofessional Team
PCCP: Primary Care Clinical Pharmacist
PCN: Primary Care Network
PCP: Primary Care Provider
UBC: University of British Columbia
US: United States
