# Supplementary material for: Unpacking the value of pharmacists in team-based primary care in British Columbia, Canada: a qualitative evaluation
Source: BMC Prim Care. 2025 Dec 9;27:58. doi: 10.1186/s12875-025-03131-2 (PMC12896227; doi:10.1186/s12875-025-03131-2)
Supplement: Supplementary file 1 — Supplementary Material 1. [file 12875_2025_3131_MOESM1_ESM.docx]

**T1 Interview Topic Guide – Executive Lead**

**Introductory/Icebreaker**

- We understand that you have been working at the Pharmacists in PCN Program as the Executive Program Lead in the past year. Can you tell us about your role in the Program?
- How has it been working with the Program so far?
- How has your experience as a pharmacist and the Associate Dean, Practice and Innovation, informed the infrastructure of the Program?

**Program Activities and Governance**

***PCCP Hiring***

- What can you tell us about the recruitment and hiring of PCCPs so far?
- Is it going as planned?
- What sort of things went smoothly and as expected during PCCP recruitment and hiring processes?
- What challenges did you face during PCCP recruitment and hiring processes?

***PCCP Onboarding***

- How does your role support the onboarding of PCCPs?
- How do you think the onboarding of PCCPs has gone so far?
- What can you tell us about the onboarding experiences of PCCPs?
- What do you think went well?
- What problems did you have to solve?

***UBC Team Onboarding***

- How does your role support the onboarding of UBC team members?
- How do you think the onboarding of UBC team members has gone so far?
- What can you tell us about the onboarding experiences of UBC team members?
- What do you think went well?
- What problems did you have to solve?

***Technical Needs and Lessons Learned***

- How have the technical needs adapted and changed as the Program grew?
- What were the learning curves? Lessons learned?

***Teamwork***

- Who do you work with the most in your role?
- What can you reflect on in terms of Program teamwork experiences?
- In your opinion, what would you consider as successes regarding interprofessional teamwork?
- On the other hand, what kind of challenges have been experienced while working on teams?

***Committees and Working Groups Engagement***

- What committees are you part of?
- Can you tell us about your role in each of these committees?
- What can you tell us about the general functioning of committees and working groups? Are they working as anticipated?
- Overall, do you feel the formal and informal committee and working group structures have effectively supported the Program implementation?

**Site Coordination and Scheduling**

***PCN Introduction/Adoption***

- Can you tell us more about the Program’s PCN integration processes?
- Can you give us examples of what you think is going well with the PCN integration processes?
- Can you provide us with an example about what a seamless introduction into a PCN looks like?
- What do think worked well with that PCN? Why?
- What do you think are factors that have enabled and supported the establishment of connections with PCNs?
- What do you think are some barriers to making connections with PCNs?
- Can you share with us any experience you had with PCN adoption hesitancy of the Program?

***PCN Coordination and Engagement***

- Generally speaking, how has your experience been so far working with the clinics across different PCNs?
- What do you think are factors that have enabled and supported The Program’s coordination with the clinics?
- How do you think the PCNs have been using UBC’s referral guidelines? How does it vary depending on model of care? Existing IPTs?
- On the other hand, what kind of issues or barriers have you faced when working with the PCNs?

***Models of Care***

- Can you explain to us the connection between different PCNs with respect to models of care?
- How has your experience been with different models of care?
- Which models of care are the best/easiest to support? Why?
- On the other hand, which ones are challenging to support? Why?

**Program Capacity**

- Do you think the Program team is working at capacity? Why/Why not?
- What can you tell us about the site coordinators/MOAs ratio to PCCPs?
- What are your thoughts/perspectives on the HR needs for site coordinators and/or MOAs for PCCPs with respect to the Program capacity?

**Community and Hospital Pharmacy Engagement**

- How well do you think the Program has been in establishing relationships between PCCPs and community pharmacists?
- How well do you think the Program has been in establishing relationships between PCCPs and hospital pharmacists?
- What are some facilitators that supported bringing PCCPs to collaborate with community and hospital pharmacists?
- What are some barriers encountered while working to bring PCCPs to collaborate with community and hospital pharmacists?

**Closing/Probing Questions**

- Where do you feel your role has the greatest impact on the Program?
- What do you think could be done to further support your role within the Program?
- What do you think are factors which facilitated implementing the Program?
- What do you think are some barriers to implementing the Program?
- What other lessons have you learned from implementing the Program so far?
- Based on your experience working within the Program, what things did you find surprising?
- Is there anything we haven’t talked about that you would like to discuss or share with us today?

**T1 Interview Topic Guide – Program Lead**

**Introductory/Icebreaker**

- We understand that you have been working at the Pharmacists in PCN Program as the Program Lead, can you explain to us your role within the Program?
- How has it been working with the Program so far?
- How has your experience as a pharmacist and the Director of the Pharmacists Clinic informed the Program operations?

**Program Activities and Governance**

***PCCP Hiring***

- What can you tell us about the recruitment and hiring of PCCPs so far?
- Is it going as planned?
- What sort of things went smoothly and as expected during PCCP recruitment and hiring process? Why?
- What challenges did you face during PCCP recruitment and hiring process? Why?
- Has anything about hiring of PCCPs challenged any aspects of Program implementation?  If so, how?

***PCCP Onboarding***

- How does your role support the onboarding of PCCPs?
- How do you think the onboarding of PCCPs has gone so far?
- What can you tell us about the onboarding experiences of PCCPs?
- What do you think went well?
- What problems did you have to solve?

***UBC Team Onboarding***

- How does your role support the onboarding of UBC team members?
- How do you think the onboarding of UBC team members has gone so far?
- What can you tell us about the onboarding experiences of UBC team members?
- What do you think went well?
- What problems did you have to solve?

***Technical Needs and Lessons Learned***

- How have the technical needs adapted and changed as the Program grew?
- What were the learning curves? Lessons learned?

***Teamwork***

- Who do you work with the most in your role?
- What can you reflect on in terms of Program teamwork experiences?
- In your opinion, what would you consider as successes regarding interprofessional teamwork?
- On the other hand, what kind of challenges has been experienced while working on teams?

***Committees and Working Groups Engagement***

- What committees are you part of?
- Can you tell us about your role in each of these committees?
- What can you tell us about the general functioning of committees and working groups? Are they working as anticipated?
- Overall, do you feel the formal and informal committee and working group structures have effectively supported the Program implementation?

**Site Coordination and Scheduling**

***PCN Introduction/Adoption***

- Can you tell us more about your involvement with the PCNs specifically the PCN integration processes?
- Can you give us examples of what you think is going well with the PCN integration processes?
- Can you provide us with an example about what a seamless introduction and integration into a PCN looks like?
- What do think worked well with that PCN? Why?
- What about the things that you think are not going well or overall have challenged integration of the PCCP into a PCN?
- What do you think are factors that have enabled and supported the Program’s relationship and PCCP integration to PCNs?
- On the other hand, what kind of issues or barriers the Program has faced when trying to establish connections with PCNs?
- Can you share with us any experiences you had with PCN adoption hesitancy of the Program?
- In your opinion, what do you think could be done to improve the establishment and integration of the Program and PCCPs with the PCNs?

***PCN Coordination and Engagement***

- Generally speaking, how has your experience been so far working with the clinics across different PCNs?
- What do you think are factors that have enabled and supported The Program’s coordination with the clinics?
- How do you think the PCNs have been using UBC’s referral guidelines/recommendations? How does it vary depending on model of care? Existing IPTs?
- On the other hand, what kind of issues or barriers have you faced when working with the PCNs?

***Models of Care***

- Can you explain to us the connection between different PCNs with respect to models of care?
- How has your experience been with different models of care?
- Which models of care are the best/easiest to support? Why?
- On the other hand, which ones are challenging to support? Why?
- How has virtual care delivery been affected by model of care, if at all?

**Program Infrastructure**

- What can you tell us about the PCN EMR coordination and interoperability within the Program?
- What do you think are factors that have enabled and supported the PCN EMR coordination across the clinics?
- On the other hand, what kind of issues or barriers have you faced regarding PCN EMR coordination across the clinics?

**Program Capacity and HR Needs**

- Do you think the Program is working at capacity? Why/Why not?
- What can you tell us about the site coordinators/MOAs ratio to PCCPs?
- What are your thoughts/perspectives on the HR needs for site coordinators and/or MOAs for PCCPs with respect to the Program capacity?

**Patient Follow-up**

- What can you tell us about patient attachment in the Program (e.g. follow-up)?
- What has been the greatest challenges to optimizing patient referrals for new patients?

**T1 Interview Topic Guide – Assistant Director, Primary Care**

**Introductory/Icebreaker**

- We understand that you have been working in the Program as the Assistant Director of Primary Care, can you explain to us your role within the Program?
- How has it been working with the Program so far?

**Program Activities and Governance**

***PCCP Hiring***

- What can you tell us about the recruitment and hiring of PCCPs so far?
- Is it going as planned?
- What sort of things went smoothly and as expected during PCCP recruitment and hiring process? Why?
- What challenges did you face during PCCP recruitment and hiring process? Why?

***PCCP Onboarding***

- How does your role support the onboarding of PCCPs?
- How do you think the onboarding of PCCPs has gone so far?
- What can you tell us about the onboarding experiences of PCCPs?
- What do you think went well?
- What problems did you have to solve?

***Teamwork***

- Who do you work with the most in your role?
- What can you reflect on in terms of Program teamwork experiences?
- In your opinion, what would you consider as successes regarding interprofessional teamwork?
- On the other hand, what kind of challenges has been experienced while working on teams?

***Committees and Working Groups Engagement***

- What committees are you part of?
- Can you tell us about your role in each of these committees?
- What can you tell us about the general functioning of committees and working groups? Are they working as anticipated?
- Overall, do you feel the formal and informal committee and working group structures have effectively supported the Program implementation?

***Technical Needs and Lessons Learned***

- How have the technical needs adapted and changed as the Program grew?
- What were the learning curves? Lessons learned?

**Community and Hospital Pharmacy Engagement**

- How well do you think the Program has been in establishing relationships between PCCPs and community pharmacists?
- How well do you think the Program has been in establishing relationships between PCCPs and hospital pharmacists?
- What are some facilitators that supported bringing PCCPs to collaborate with community and hospital pharmacists?
- What are some barriers encountered while working to bring PCCPs to collaborate with community and hospital pharmacists?

**Site Coordination and Scheduling**

***PCN Introduction/Adoption***

- Can you tell us more about your involvement with the PCNs specifically the PCN integration processes?
- Can you give us examples of what you think is going well with the PCN integration processes?
- Can you provide us with an example about what a seamless introduction into a PCN looks like?
- What do think worked well with that PCN? Why?
- What about the things that you think are not going well?
- Based on your experience working in the Program, what tends to work well with PCN introduction?
- What do you think are factors that have enabled and supported the establishment of a relationship with PCNs?
- On the other hand, what kind of issues or barriers have you faced when trying to establish a relationship with PCNs?
- Can you share with us any experience you had with PCN adoption hesitancy of the Program?
- In your opinion, what do you think could be done to improve the introduction and relationship of the Program within PCNs?

***PCN Coordination and Engagement***

- Generally speaking, how has your experience been so far working with the clinics across different PCNs?
- What do you think are factors that have enabled and supported The Program’s coordination with the clinics?
- How do you think the PCNs have been using UBC’s referral guidelines? How does it vary depending on model of care? Existing IPTs?
- On the other hand, what kind of issues or barriers have you faced when working with the PCNs?

***Models of Care***

- Can you explain to us the connection between different PCNs with respect to models of care?
- How has your experience been with different models of care?
- Which models of care are the best/easiest to support? Why?
- On the other hand, which ones are challenging to support? Why?

***Program Infrastructure***

- What can you tell us about the EMR coordination and interoperability within the Program?
- What do you think are factors that have enabled and supported EMR coordination with clinics across the PCNs?
- On the other hand, what kind of issues or barriers have you faced regarding EMR coordination with clinics across the PCNs?

***Program Capacity***

- Do you think the Program is working at capacity? Why/Why not?
- What can you tell us about the site coordinators/MOAs ratio to PCCPs?
- What are your thoughts/perspectives on the HR needs for site coordinators and/or MOAs for PCCPs with respect to the Program capacity?

**Closing/Probing Questions**

- Where do you feel your role has the greatest impact on the Program?
- What do you think could be done to further support your role within the Program?
- What do you think are factors which facilitated implementing the Program?
- What do you think are some barriers to implementing the Program?
- What other lessons have you learned from implementing the Program so far?
- Based on your experience working within the Program, what things did you find surprising?
- Is there anything we haven’t talked about that you would like to discuss or share with us today?

**T1 Interview Topic Guide – Coordinator, Primary Care**

**Introductory/Icebreaker**

- We understand you have been working at the Pharmacists in PCN Program as the Coordinator, Primary care, can you explain to us your role within the Program?
- How has it been working with the Program so far?

**Community Engagement with Pharmacists**

- Can tell us what happens during the initial activities such as relationship building?
- Can you give us examples of what you think is going well with introducing the Program to community pharmacy stakeholders?
- What about the things that you think are not going well?
- What do you think are factors that have enabled and supported the engagement with community pharmacy stakeholders?
- Does the quality of connection/process of relationship building differ if it is a community pharmacist or HA pharmacist?
- On the other hand, what kind of issues or barriers have you faced when trying to connect with community pharmacy stakeholders?
- Can you walk us through the engagement path with each PCCP?
- Based on your experience working within the Program, what are your impressions of initial success of community pharmacy engagement and Program introduction?
- In your opinion, what do you think could be done to improve the engagement with community pharmacy stakeholders and Program introduction?

**Program Activities and Governance**

***Teamwork***

- Who do you work with the most in your role?
- What can you reflect on in terms of Program teamwork experiences?
- In your opinion, what would you consider as successes regarding interprofessional teamwork?
- On the other hand, what kind of challenges has been experienced while working on teams?

***Committees and Working Groups Engagement***

- What committees are you part of?
- Can you tell us about your role in each of these committees?
- What can you tell us about the general functioning of committees and working groups? Are they working as anticipated?
- Overall, do you feel the formal and informal committee and working group structures have effectively supported the Program implementation?

***Technical Needs and Lessons Learned***

- How have the technical needs adapted and changed as the Program grew?
- What were the learning curves? Lessons learned?

**Program Capacity**

- Do you think the program is working at capacity? Why/Why not?
- What can you tell us about the site coordinators/MOAs ratio to PCCPs?
- What are your thoughts/perspectives on the HR needs for site coordinators and/or MOAs for PCCPs with respect to the Program capacity?

**Probing/Closing Questions**

- Where do you feel your role has the greatest impact on the Program?
- What do you think could be done to further support your role within the Program?
- What do you think are factors which facilitated implementing the Program?
- What do you think are some barriers to implementing the Program?
- What other lessons have you learned from implementing the Program so far?
- Based on your experience working within the Program, what things did you find surprising?
- Is there anything we haven’t talked about that you would like to discuss or share with us today?

**T1 Interview Topic Guide – Coordinator, Quality Care**

**Introductory/Icebreaker**

- We understand you have been working at the Pharmacists in PCN Program as the Coordinator, Quality Care can you explain to us your role within the Program?
- How has it been working with the Program so far?

**Program Activities and Governance**

***PCCP Hiring***

- What are you looking for when hiring a PCCP? Previous experiences?
- What can you tell us about the recruitment and hiring of PCCPs so far?
- Is it going as planned?
- What sort of things went smoothly and as expected during PCCP recruitment and hiring process? Why?
- What challenges did you face during PCCP recruitment and hiring process? Why?

***PCCPs Onboarding***

- How does your role support the onboarding of PCCPs?
- How do you think the onboarding of PCCPs has gone so far?
- What can you tell us about the onboarding experiences of PCCPs?
- What do you think went well?
- What problems did you have to solve?
- What are some barriers encountered while working to bring PCCPs into the community?

***Teamwork***

- Who do you work with the most in your role?
- What can you reflect on in terms of Program teamwork experiences?
- What are some elements of collaboration to ensure the integration of PCCPs into teams?
- In your opinion, what would you consider as successes regarding interprofessional teamwork?
- On the other hand, what kind of challenges has been experienced while working on teams?

***Technical Needs and Lessons Learned***

- How have the technical needs adapted and changed as the Program grew?
- What were the learning curves? Lessons learned?

***PACT Program***

- We understand that part of your role is to develop and deliver the PACT Program. What can you tell us about that?
- Is the enrolment and completion going as expected?
- What activities have you facilitated to build a community of practice?

***Committees and Working Groups Engagement***

- Which committees are you a part of?
- Can you tell us about your role in each of these committees?
- What can you tell us about the general functioning of committees and working groups? Are they working as anticipated?
- Overall, do you feel the formal and informal committee and working group structures have effectively supported the Program implementation?

**Engagement with Community and Hospital Pharmacists**

- Have you had any experience working with community and hospital pharmacists?
- Can you talk about it more? What did you do?
- How frequent do you interact with them?
- How well do you think the Program has been in establishing relationships between PCCPs and community pharmacists?
- How well do you think the Program has been in establishing relationships between PCCPs and hospital pharmacists?
- What are some facilitators that supported bringing PCCPs to collaborate with community and hospital pharmacists?
- What are some barriers encountered while working to bring PCCPs to collaborate with community and hospital pharmacists?

***Program Infrastructure***

- Can you tell us about your experiences with Clinical and EMR troubleshooting and they have supported coordination of care (facilitators and barriers)?

***Program Capacity***

- Do you think the program is working at capacity? Why/Why not?
- What can you tell us about the site coordinators/MOAs ratio to PCCPs?
- What are your thoughts/perspectives on the HR needs for site coordinators and/or MOAs for PCCPs with respect to the Program capacity?

**Site Coordination and Scheduling**

- Can you tell us more about your involvement with the PCNs specifically the PCN integration processes?
- Can you give us examples of what you think is going well with the PCN integration processes?
- What about the things that you think are not going well?

**Referral Guidelines**

- What referral guidelines have been generated by the Program? What determines how the PCN GPs/NPs using them?
- How much do you think the PCNs have been using them?

**Quality Care**

- We understand that commitment to quality of care involves discussing patient cases with PCCPs pre and post appointment in addition to reviewing and providing feedback on their documentation, can you walk us through what happens during these processes?
- Can you share with us your experience with education discussions?
- What can you tell us about the results of your performance/peer assessments?
- Do your assessments include observing instances of shared decision making? What can you tell us about that?
- What aspects of the Program support your role the most?
- What challenges have you faced during this role?
- How has virtual care delivery affected quality of care of complex patients and their families? How did it vary by model of care?
- How does the Program ensure that the PCCP’s time has been best utilized to provide patient care?
- What kind of barriers or challenges were faced during this process?
- What was the Program’s approach to address them?

**Patient Follow-up**

- What can you tell us about patient attachment in the program (e.g. follow-up)?
- Can you tell us about some experiences of patients following up with the Program after hospitalization?

**Probing/Closing Questions**

- Where do you feel your role has the greatest impact on the Program?
- What do you think could be done to further support your role within the Program?
- What do you think are factors which facilitated implementing the Program?
- What do you think are some barriers to implementing the Program?
- What other lessons have you learned from implementing the Program so far?
- Based on your experience working within the Program, what things did you find surprising?
- Is there anything we haven’t talked about that you would like to discuss or share with us today?

**T1 Interview Topic Guide – Site Coordinator Team Lead**

**Introductory/Icebreaking**

- We understand that you have been working at the Pharmacists in PCN Program as the site coordination team lead, can you explain to us your role within the Program?
- How has it been working with the Program so far?

**Program Activities and Governance**

***PCCP Onboarding***

- How does your role support the onboarding of PCCPs?
- How do you think onboarding of PCCPs has gone so far?
- What can you tell us about the onboarding experiences of PCCPs?
- What do you think went well?
- What problems did you have to solve?

***UBC Team Onboarding***

- How does your role support the onboarding of UBC team members?
- How do you think onboarding of UBC team members has gone so far?
- What can you tell us about the onboarding experiences of UBC team members?
- What do you think went well?
- What problems did you have to solve?

***Teamwork***

- Who do you work with the most in your role stakeholders or team members?
- What can you reflect on in terms of Program teamwork experiences?
- In your opinion, what would you consider as successes regarding interprofessional teamwork?
- On the other hand, what kind of challenges has been experienced while working on teams?

***Committees and Working Groups Engagement***

- What committees are you a part of?
- Can you tell us about your role in each of these committees?
- In your opinion, how well do you think these committees are functioning?

**Site Coordination and Scheduling**

***PCN Introduction/Adoption***

- Can you tell us more about the Program’s PCN integration processes?
- Can you give us examples of what you think is going well with the PCN integration processes?
- Can you provide us with an example about what a seamless introduction into a PCN looks like?
- What do think worked well with that PCN? Why?
- What do you think are factors that have enabled and supported the establishment of connections with PCNs?
- What do you think are some barriers to making connections with PCNs?
- Can you share with us any experience you had with PCN adoption hesitancy of the Program?

***PCN Coordination and Engagement***

- Generally speaking, how has your experience been so far working with the clinics across different PCNs?
- What do you think are factors that have enabled and supported The Program’s coordination with the clinics?
- How do you think the PCNs have been using UBC’s referral guidelines? How does it vary depending on model of care? Existing IPTs?
- On the other hand, what kind of issues or barriers have you faced when working with the PCNs?

***Program Referral and Scheduling Processes***

- Can you tell us about the different referral paths for patients to see a PCCP? How do they vary based on model of care? PCN?
- What do you think affect the referral paths to the Program?
- Can you tell us about different ways of scheduling patient appointments and how they differ according to models of care?

***Models of Care***

- Can you explain to us the connection between different PCNs with respect to models of care?
- How has your experience varied among the three models of care?
- Which models of care are the best/easiest to support? Why?
- On the other hand, which ones are challenging to support? Why?
- How has virtual care delivery varied by model of care?

***PCN Location and Health Authorities***

- Can you share with is your experiences with rural vs urban PCNs?
- What about your experiences across different health authorities?

***Referral Volumes and Program Capacity***

- In your opinion, what are the factors affecting the referral volumes?
- What is the relation between referral volumes and models of care?
- What has been done so far to optimize the referral volumes?
- What can you tell us about the site coordinators ratio to PCCPs?
- What are your thoughts/perspectives on the HR needs for site coordinators for PCCPs with respect to the Program capacity?

***EMR and Case Conference Coordination***

- What can you tell us about the EMR coordination and interoperability within the Program?
- What do you think are factors that have enabled and supported EMR coordination with the clinics across the PCNs?
- On the other hand, what kind of issues or barriers have you faced regarding EMR coordination across the clinics?
- Can you explain to us your experience with case conference coordination?
- What do you think are some factors that support your role in coordinating case conferences?

***Patient Follow-up***

- What can you tell us about patient attachment in the program (e.g. follow-up)?
- Can you tell us about some experiences of patients following up with the Program after hospitalization?

**Closing/Probing Questions**

- Where do you feel your role has the greatest impact on the Program?
- What do you think could be done to further support your role within the Program?
- What do you think are factors which facilitated implementing the Program?
- What do you think are some barriers to implementing the Program?
- What other lessons have you learned from implementing the Program so far?
- Based on your experience working within the Program, what things did you find surprising?
- Is there anything we haven’t talked about that you would like to discuss or share with us today?

**T1 Focus Group Topic Guide – Primary Care Clinical Pharmacists (PCCPs)**

**Introductory/Icebreaker**

- We understand you have been working as a PCCP within the Pharmacists in PCN Program, how has it been working with the Program so far?
- How has it changed over time?

**Hiring and Onboarding**

- What can you tell us about your experiences with the hiring process?
- What can you tell us about your experiences with the UBC-based onboarding, specifically the first week?
- What can be done to improve the hiring process?
- What can be done to improve the onboarding experience of PCCPs?

**Assessments and Continuing Training (Quality Care)**

- What have your experiences been with the assessment aspect of the Program?
- What have your experiences been with the professional development aspects of the Program?

**Care Delivery**

- Can you reflect the different models of care in terms of their advantages and disadvantages?
- What can you tell us about your experiences with the technical and connectivity aspects of the Program?
- How has it affected the delivery of care?
- How well has UBC supported you in delivering care within your model of care?
- How well has the clinic that you work with supported you with this model of care? [if applicable]
- Tell us about the extent to which you are able to have latitude and influence in clinical decision making that supports your patients, specifically in resolving DTPs?
- Do you think virtual appointments have affected quality of care, if at all?

**Experiences Working with Complex Patients and Their Families**

- What can you tell us about a typical patient that you see in terms of complexity?
- How often do you care for patients with a family member present?
- What are your experiences with this from a clinical advising perspective?
- What are your experiences from a coordination perspective?
- How do you think your model of care affects working with complex patients and their families?

**Shared Decision Making**

- How has the Program supported your role in the SDM process?
- How do you think the Program could better support your role in the SDM process?

**Experiences Working with the UBC Team**

- How has your experience been working with the Program team at UBC?
- How has your experience been working with the Program’s Pharmacy Leads?

**Experiences Working with other Primary Care Providers (Family Physicians/Nurse Practitioners)**

- What role did the patient play in cross-communication with the FP/NP?
- What are some differences that you have observed with different types of care provider practices?
- How effective has the Program been in coordinating your interactions with the FP/NP?
- How do you think your model of care affects interaction with a FP/NP?

**Experiences Working with other IPT members**

- Tell us about your experiences working with an allied health care professional within the PCNs?
- What role did the patient play in cross-communication with the IPT member?
- What has been the role of the Program in coordinating your interactions with the IPT members?
- How do you think your model of care affects interaction with IPT members?

**Experiences Working with Community and Hospital Pharmacists**

- Can you share with us your experiences connecting with community pharmacists?
- What role did the patient play in the cross-communication with the community pharmacist?
- What role did the Program play in coordinating your interactions with the community pharmacist?
- How was your experience been working with transitions of care with hospital pharmacists in the Program?
- What else can you say about working with community or hospital pharmacists in your role?

**Referral Volumes**

- In your opinion, what are the factors affecting the referral volumes?
- What do you think could be done to optimize referrals?

**Overall Experiences of Program**

- What are some factors that have an impact on your satisfaction with your current role as a PCCP?
- We understand that you have been advocating for your role as a PCCP within the PCNs, what can you tell us about this?
- What do you think could be done to further support your role within the Program?
- What lessons have you learned from working within the Program so far?
- Based on your experience working within the Program, what surprised you the most about your role or the Program so far?
- Is there anything that you would like to say that was not already covered by these questions?

**T2 Interview Topic Guide – Executive Lead**

**Introductory/Icebreaker**

- How has your role as Executive Lead evolved throughout the course of the Program?

**PCN Introduction/Adoption**

- Can you share with us any experience you had with PCN adoption hesitancy of the Program during the third year?
- What do you think about the overall readiness for adopting PCCPs into PCN? How does it compare to the first two years of the Program?

**Program and PCN Infrastructure for Integration**

- Have centralized resources, systems and processes been optimal and effective to support Program implementation? What can you tell us about this?
- What have been the most/least utilized resources to support clinical care in the PCNs?
- Can you tell us more about the Program’s PCN integration processes throughout?
- How have the enabling factors which supported that establishment of PCN connections changed throughout the course of the Program?
- How do you think the barriers to making connections with PCNs have evolved during the course of the Program? (space issues, Wi-Fi, technical challenges, EMR access)
- What PCN infrastructure factors have been enablers/barriers to PCCP integration?
- What activities have the Program led to continuously engage with PCNs and how effective have they been?

**Program Governance and Stakeholder Engagement**

- How has the Program’s governance model been effective to support implementation?
- How have the challenges faced by the leadership team at the decision-making level evolved over the last year and as we are getting closer to the Program transition?
- What committees and working groups were essential to the implementation and should be considered for the sustainability or expansion of the Program?
- What additional resources do you think would have supported Program implementation?

**Communications and Reporting**

- What are your thoughts on the Program’s communication plan? Do you think is has been executed as planned?
- What was the impact of the formal and informal communications and reporting for the Program the target audiences? Do you think they have been timely and useful to the target audiences?

**Referral Volumes and Program Capacity**

- How have the appointment volumes evolved over the course of the Program?
- How beneficial were the measures taken by the Program to optimize the referral volumes?
- What were the specific approaches for increasing patient referrals that had the biggest impact on patient appointments?
- What did work and did not work?

**EMR Coordination and Access**

- How have the issues/barriers faced regarding EMR coordination across the clinics evolved throughout the Program (access and permissions to utilize EMR)?
- What changed and what improvements were made?
- How have the challenges of some PCCPs accessing Pharmanet evolved to optimize MOAs’ and PCCPs’ role? How has this barrier been addressed since last year?

**Patient Follow-up**

- What can you tell us about patient attachment in the program (e.g., follow-up)?
- Can you tell us about some experiences of patients following up with the Program after hospitalization? How are they different from the first two years of the Program?

**Intraprofessional Collaboration**

- What is the extent and nature of the interactions between a PCCP and other pharmacists? What has been the nature and frequency of the information exchange?
- What processes, systems and workflows have been identified as enablers of collaboration?
- What processes, systems and workflows have been identified as barriers to collaboration?

**Technical Needs and Lessons Learned**

- How have the technical needs adapted and changed throughout the Program? What are the lessons learned?

**PCN Location and Health Authorities**

- Can you share with us your experiences with rural vs urban PCNs compared to the first two years?
- What about your experiences across different health authorities compared to the first two years?

**Program Transition**

***UBC Supporting Resources***

- What are the critical elements of the Program at the PCN level that should be retained at the time of transition?
- If the Program was to run for 3 years more as we know it now, what do you think that could be done differently to enhance its implementation?

If you could think of resources that UBC offered to support Program implementation such as HR (MOAs, Site Coordinators and Quality Care team) and technological support, which elements of UBC central support do you think PCNs would need after Program transition?

- What other lessons have you learned from implementing the Program so far and what do you think should be considered for its transition?
- For the Program elements you know/expect to be discontinued, could you elaborate on the level of impact you predict on the Program?
- What do you anticipate as potential barriers and issues post-transition?
- What do you think have been the sources of frustrations during the last 6 months of the transition in respect to organization, communications, and governance?
- Do you think the new proposed structure (what is known so far) can be expected to maintain the Program in the future?
- What are your thoughts/perspectives on the HR needs for site coordinators for PCCPs after Program transition?
- We understand the Ministry has made a decision to not continue UBC MOAs in their role post transition. What are your thoughts on that?

***PCN-Related Factors***

- What factors do you think would affect adoption readiness of PCNs?
- How will PCN acceptability affect transferability of the Program?
- How do you think scalability will differ within each PCN?

***EMR Transferability***

- How will OSCAR EMR centralization/interoperability and scheduling be affected in each PCN after transition?

**Probing/Closing Questions**

- What are your thoughts on the future pathway for pharmacy training in primary care?
- What are the advantages of central Program delivery by UBC?
- What are the disadvantages of central Program delivery by UBC?
- Where do you feel your role had the greatest impact on the Program?
- What do you think could have been done to further support your role within the Program?
- What do you think are factors which facilitated implementing the Program?
- What do you think are some barriers to implementing the Program?
- What other lessons have you learned from implementing the Program so far?
- Based on your experience working within the Program, what things did you find surprising?
- Is there anything we haven’t talked about that you would like to discuss or share with us today

**T2 Interview Topic Guide – Program Lead**

**Introductory/Icebreaker**

- How has your role as the Assistant Director and then as Program Lead evolved throughout the course of the Program?

**PCN Introduction/Adoption**

- Can you share with us any experience you had with PCN adoption hesitancy of the Program during the third year?
- What do you think about the overall readiness for adopting PCCPs into PCN? How does it compare to the first two years of the Program?

**Program and PCN Infrastructure for Integration**

- Have centralized resources, systems and processes been optimal and effective to support Program implementation? What can you tell us about this?
- What has been the most/least utilized resources to support clinical care in the PCNs?
- Can you tell us more about the Program’s PCN integration processes throughout?
- How have the enabling factors which supported that establishment of PCN connections changed throughout the course of the Program?
- How do you think the barriers to making connections with PCNs have evolved during the course of the Program? (space issues, Wi-Fi, technical challenges, EMR access)
- What PCN infrastructure factors have been enablers/barriers to PCCP integration?
- What activities have the Program led to continuously engage with PCNs and how effective has it been?

**Intraprofessional Collaboration**

- What is the extent and nature of the interaction between a PCCP and other pharmacists? What has been the nature and frequency of the information exchange?
- What processes, systems and workflows have been identified as enablers of collaboration?
- What processes, systems and workflows have been identified as barriers to collaboration?

**Needs and Lessons Learned**

- How have the technical needs adapted and changed throughout the Program? What are the lessons learned?

**PCN Location and Health Authorities**

- Can you share with us your experiences with rural vs urban PCNs compared to the first two years?
- What about your experiences across different health authorities compared to the first two years?

**Referral Volumes and Program Capacity**

- How have the appointment volumes evolved over the course of the Program?
- How beneficial were the measures taken by the Program to optimize the referral volumes?
- What were the specific approaches for increasing patient referrals that had the biggest impact on appointment Volumes?
- What did work and did not work?

**EMR Coordination and Access**

- How have the issues/barriers faced regarding EMR coordination across the clinics evolved throughout the Program (access and permissions to utilize EMR)?
- What changed and what improvements were made?
- How have the challenges of some PCCPs accessing Pharmanet evolved o to optimize MOAs’ and PCCPs’ role? How has this barrier been addressed since last year?

**Patient Follow-up**

- What can you tell us about patient attachment in the Program (e.g. follow-up)?
- Can you tell us about some experiences of patients following up with the Program after hospitalization? How are they different from the first two years of the Program?

**Program Governance and Stakeholder Engagement**

- How has the Program’s governance model been effective to support implementation?
- How have the challenges faced by the leadership team at the decision-making level evolved over the last year and as we are getting closer to the Program transition?
- What committees and working groups were essential to the implementation and should be considered for the sustainability or expansion of the Program?

**Communications and Reporting**

- What are your thoughts on the Program’s communication plan? Do you think is has been executed as planned?
- What was the impact of the formal and informal communications for the Program the target audiences? Do you think they have been timely and useful to the target audiences?

**Program Transition**

***UBC Supporting Resources***

- What are the critical elements of the Program at the PCN level that should be retained at the time of transition?
- If the Program was to run for 3 years more as we know it now, what do you think could be done differently to enhance its implementation?
- If you could think of resources that UBC offered to support Program implementation such as HR (MOAs, Site Coordinators and Quality Care team) and technological support, which elements of UBC central support do you think PCNs would need after Program transition?
- What other lessons have you learned from implementing the Program so far and what do you think should be considered for its transition?
- For the program elements you know/expect to be discontinued, could you elaborate on the level of impact you predict on the Program?
- What do you anticipate as potential barriers and issues post-transition?
- What do you think have been the sources of frustrations during the last 6 months of the transition in respect to organization, communications, and governance?
- Do you think the new proposed structure (what is known so far) can be expected to maintain the Program in the future?
- What are your thoughts/perspectives on the HR needs for site coordinators for PCCPs after Program transition?
- We understand the Ministry has made a decision to not continue UBC MOAs in their role post transition, what are your thoughts on that?

***PCN-Related Factors***

- What factors do you think would affect adoption readiness of PCNs?
- How will PCN acceptability affect transferability of the Program?
- How do you think scalability will differ within each PCN?

***EMR Transferability***

- How will OSCAR EMR centralization/interoperability and scheduling be affected in each PCN after transition?

**Probing/Closing Questions**

- What are your thoughts on the future pathway for pharmacy training in primary care?
- What are the advantages of central Program delivery by UBC?
- What are the disadvantages of central Program delivery by UBC?
- Where do you feel your role had the greatest impact on the Program?
- What do you think could have been done to further support your role within the Program?
- What do you think are factors which facilitated implementing the Program?
- What do you think are some barriers to implementing the Program?
- What other lessons have you learned from implementing the Program so far?
- Based on your experience working within the Program, what things did you find surprising?
- Is there anything we haven’t talked about that you would like to discuss or share with us today?

**T2 Interview Topic Guide – Primary Care Coordinator**

**Introductory/Icebreaker**

- How has your role as a Coordinator, Primary Care evolved throughout the course of the Program?

**Community Engagement with Pharmacists**

- Can you give us examples of what you think is going well with introducing the Program to community pharmacy stakeholders **compared to the first two years**?
- What about the things that you think are not going well?
- What do you think are factors that have enabled and supported the engagement with community pharmacy stakeholders so far?
- On the other hand, what kind of issues or barriers have you faced when trying to connect with community pharmacy stakeholders **compared to the first two years**?
- Based on your experience working within the Program, what are your overall impressions of successes of community pharmacy engagement and Program introduction?
- In this integration of care between PCCPs and community pharmacists, can you speak to any feedback received about patient perceptions and experiences?
- In your opinion, what do you think could be done to improve PCCPs’ engagement with community pharmacy stakeholders and Program introduction **post-transition**?

**Intraprofessional Collaboration**

- What is the extent and nature of the interaction between a PCCP and other pharmacists? What has been the nature and frequency of the information exchange?
- What processes, systems and workflows have been identified as enablers of collaboration?
- What processes, systems and workflows have been identified as barriers to collaboration?
- With the collaboration of PCCPs and other pharmacists, can you speak to its overall impact you have seen on the quality of care in the last 3 years?

**Technical Needs and Lessons Learned**

- How have the technical needs adapted and changed throughout the Program? What are the lessons learned?

**Program Transition**

***UBC Supporting Resources***

- What are the critical elements of the Program at the PCN level that should be retained at the time of transition?  If you could think of resources that UBC offered to support Program implementation such as HR (MOAs, Site Coordinators and Quality Care team) and technological support, which elements of UBC central support do you think PCNs would need after Program transition
- If the Program was to run for 3 years more as we know it now, what do you think that could be done differently to enhance its implementation?
- What other lessons have you learned from implementing the Program so far and what do you think should be considered for its transition?
- For the Program elements you know/expect to be discontinued, could you elaborate on the level of impact you predict on the Program?
- What do you anticipate as potential barriers and issues post-transition?
- What do you think have been the sources of frustrations during the last 6 months of the transition in respect to organization, communications, and governance?
- Do you think the new proposed structure (what is known so far) can be expected to maintain the Program in the future?
- What are your thoughts/perspectives on the HR needs for site coordinators for PCCPs after Program transition?
- We understand the Ministry has made a decision to not continue UBC MOAs in their role post transition. What are your thoughts on that?

***EMR Transferability***

- How will OSCAR EMR centralization/interoperability and scheduling be affected in each PCN after transition?

**Program Governance and Stakeholder Engagement**

- How has the Program’s governance model been effective to support implementation?
- How have the challenges faced by the leadership team at the decision-making level evolved over the last year and as we are getting closer to the Program transition?
- What committees and working groups were essential to the implementation and should be considered for the sustainability or expansion of the Program?
- What additional resources do you think would have supported Program implementation?

**Probing/Closing Questions**

- What are your thoughts on the future pathway for pharmacy training in primary care?
- What are the advantages of central Program delivery by UBC?
- What are the disadvantages of central Program delivery by UBC?
- Where do you feel your role had the greatest impact on the Program?
- What do you think could have been done to further support your role within the Program?
- What do you think are factors which facilitated implementing the Program?
- What do you think are some barriers to implementing the Program?
- What other lessons have you learned from implementing the Program so far?
- Based on your experience working within the Program, what things did you find surprising?
- Is there anything we haven’t talked about that you would like to discuss or share with us today?

**T2 Interview Topic Guide – Site Coordinator Team Lead**

**Introductory/Icebreaker**

- How has your role as a Site Coordinator Team Lead evolved throughout the course of the Program?

**PCN Introduction/Adoption**

- Can you share with us any experience you had with PCN adoption hesitancy of the Program during the third year?
- What do you think about the overall readiness for adopting PCCPs into PCN? How does it compare to the first two years of the Program?

**Program and PCN Infrastructure for Integration**

- Have centralized resources, systems and processes been optimal and effective to support Program implementation? What can you tell us about this?
- What has been the most/least utilized resources to support clinical care in the PCNs?
- Can you tell us more about the Program’s PCN integration processes throughout?
- How have the enabling factors which supported that establishment of PCN connections changed throughout the course of the Program?
- How do you think the barriers to making connections with PCNs have evolved during the course of the Program?
- What PCN infrastructure factors have been enablers/barriers to PCCP integration?
- What activities have the Program led to continuously engage with PCNs and how effective has it been?

**PCN Coordination and Engagement**

- How have the enabling factors which supported The Program’s coordination with the clinics changed throughout the Program?
- On the other hand, how have the issues or barriers faced when working with the PCNs evolved throughout the Program?
- How do you think the PCNs have been using UBC’s referral guidelines? How does it vary depending on model of care? Existing IPTs?

**Intraprofessional Collaboration**

- What was the impact of these processes on PCCPs uptake/patient appointments?
- What is the extent and nature of the interaction between a PCCP and other pharmacists? What has been the nature and frequency of the information exchange?
- What processes, systems and workflows have been identified as enablers of collaboration?
- What processes, systems and workflows have been identified as barriers to collaboration?

**Technical Needs and Lessons Learned**

- How have the technical needs adapted and changed throughout the Program? What are the lessons learned?

**Models of Care**

- How has your experience varied among the three models of care?
- How does the transferability of models of care differ between each other? Are some models of care easier to transfer than others?
- What do you think are some enablers of implementing a colocation model?
- Why do you think there is still challenges to implementing a colocation model?

**PCN Location and Health Authorities**

- Can you share with us your experiences with rural vs urban PCNs compared to the first two years?
- What about your experiences across different health authorities compared to the first two years?

**Referral Volumes and Program Capacity**

- How have the appointment volumes evolved over the course of the Program?
- How beneficial were the measures taken by the Program to optimize the referral volumes?
- What were the specific approaches for increasing patient referrals that had the biggest impact on appointment volumes?
- What did work and did not work?

**EMR Coordination**

- How have the issues/barriers faced regarding EMR coordination across the clinics evolved throughout the Program (access and permissions to utilize EMR)?
- What changed and what improvements were made?

**Patient Follow-up**

- What can you tell us about patient attachment in the Program (e.g. follow-up)?
- Can you tell us about some experiences of patients following up with the Program after hospitalization? How are they different from the first two years of the Program?

**Program Transition**

***UBC Supporting Resources***

- What are the critical elements of the Program at the PCN level that should be retained at the time of transition?
- If the Program was to run for 3 years more as we know it now, what do you think that could be done differently to enhance its implementation?
- If you could think of resources that UBC offered to support Program implementation such as HR (MOAs, Site Coordinators and Quality Care team) and technological support, which elements of UBC central support do you think PCNs would need after Program transition
- What other lessons have you learned from implementing the Program so far and what do you think should be considered for its transition?

***PCN-Related Factors***

- What factors do you think would affect adoption readiness of PCNs?
- How will PCN acceptability affect transferability of the Program?
- How do you think scalability will differ within each PCN?

***EMR Transferability***

- How will OSCAR EMR centralization/interoperability and scheduling be affected in each PCN after transition?

**Probing/Closing Questions**

- What are the advantages of central Program delivery by UBC?
- What are the disadvantages of central Program delivery by UBC?
- Where do you feel your role had the greatest impact on the Program?
- What do you think could have been done to further support your role within the Program?
- What do you think are factors which facilitated implementing the Program?
- What do you think are some barriers to implementing the Program?
- What other lessons have you learned from implementing the Program so far?
- Based on your experience working within the Program, what things did you find surprising?
- Is there anything we haven’t talked about that you would like to discuss or share with us today?

**T2 Focus Group Topic Guide – Quality Care Team**

**Introductory/Icebreaker**

- How has your role in the Quality Care team evolved throughout the course of the Program?
- What aspects of the Program have supported your role the most so far? What has changed compared to the beginning of the Program?

**Quality Care**

- We understand that commitment to quality of care involves discussing patient cases with PCCPs pre and post appointment in addition to reviewing and providing feedback on their documentation, can you tell us how this process has evolved during the course of the Program?
- What has been the PCCP experience on the extent of PCN readiness to intergrade a PCCP into the PCN IPT?
- Can you share with us your experience with education discussions so far?
- In what ways have the performance/peer assessment been supportive to the PCCP so far?
- How has virtual care delivery affected quality of care of complex patients and their families during the course of the Program? How has it varied by model of care?
- What kind of barriers or challenges have been faced during this process so far? What has been the Program’s approach to address them?
- What barriers still remain until this point?
- How has the Program ensured that the PCCP’s time has been best utilized to provide patient care?

**PACT Program**

- We understand that part of your role is to develop and deliver the PACT Program. How has it been so far?
- Has the Program been delivered in a timely way?
- Has the enrolment and completion going as expected so far?
- How has the interest been among community pharmacists compared to the initial phase of the PACT Program?
- What new/ different activities have you facilitated to build a community of practice if any?
- What can you tell us about the evidence of increased PACT interest and enrolment by community pharmacists?
- What are your thoughts on the PACT Program continuing post-Program transition/future? Value?
- What do you think are some measures to increase the uptake of PACT Program in the future?

**Program and PCN Infrastructure for Integration**

- How have the facilitators for the Clinical and EMR troubleshooting changed in the past 12 months?
- How have the barriers for the Clinical and EMR troubleshooting evolved in the past 12 months? What barriers remain?
- Have centralized resources, systems and processes been optimal and effective to support Program implementation? What can you tell us about this?
- What do you think that needs to be done to improve this aspect?
- What has been the most/least utilized resources to support clinical care in the PCNs?
- Can you tell us more about the Program’s PCN integration processes throughout?
- What PCN infrastructure factors have been enablers/barriers to PCCP integration?
- How have the enabling factors which supported that establishment of PCN connections changed throughout the course of the Program?
- How do you think the barriers to making connections with PCNs have evolved during the course of the Program?
- What activities have the program led to continuously engage with PCNs and how effective has it been?

**PCN Readiness and Support**

- What do you think about the overall readiness for adopting PCCPs into PCNs? How does it compare to the first two years of the Program?
- What is a good way to assess readiness for a PCN to adopt PCCPs into their system or team?
- In your opinion, what could be done to improve integration of PCCPs into primary care?

**Technical Needs and Lessons Learned**

- How have the technical needs adapted and changed as the Program grew?
- What have been new learning curves? What have been the lessons learned?

**Teamwork/Interprofessional Collaboration**

- What processes have UBC put in place to raise awareness about having a PCCP in the PCN and prepare for shared care of mutual patients between PCCPs and other pharmacists in each PCN community (community/hospital)?
- What was the impact of these processes on PCCPs uptake/patient appointments?
- What is the extent and nature of the interaction between a PCCP and other pharmacists? What has been the nature and frequency of the information exchange?
- What processes, systems and workflows have been identified as enablers of collaboration?
- How have these successes evolved throughout Program?
- What processes, systems and workflows have been identified as barriers to collaboration?
- How have these barriers evolved throughout Program?
- What has been the impact on the quality of care when collaboration between a PCCP and another pharmacist occurs?

**Engagement with Community and Hospital Pharmacists**

- Can you share your experiences working with community and hospital pharmacists throughout the Program?
- How frequent do you interact with them?
- Overall, how well do you think the Program has been in establishing relationships between PCCPs and **community pharmacists** during its course?
- Overall, how well do you think the Program has been in establishing relationships between PCCPs and **hospital pharmacists** during its course?
- How have facilitators that supported bringing PCCPs to collaborate with community and hospital pharmacists changed during the course of the Program?
- How have barriers encountered while working to bring PCCPs to collaborate with community and hospital pharmacists changed during the course of the Program?

**Patient Follow-up**

- What can you tell us about patient attachment in the Program (e.g. follow-up) so far?
- Can you tell us about some experiences of patients following up with the Program after hospitalization?
- What measures does your team implement to ensure quality care in patient follow-up appointments to resolve DTPs and optimize patient care?

**Program Transition**

***UBC Supporting Resources***

- What are the critical elements of the Program that should be retained at the time of transition? (At a PCN level)
- What will be the impact some of the Program supporting elements should they not continue following transition?
- If you could think of resources that UBC offered to support Program implementation such as HR (MOAs, Site Coordinators and Quality Care team) and technological support, which elements of UBC central support do you think PCNs would need after Program transition?
- What other lessons have you learned from implementing the Program so far and what do you think should be considered for its transition?

***PCN-Related Factors***

- What factors do you think would affect adoption readiness of PCNs?
- How will PCN acceptability affect transferability of the Program?
- How do you think scalability will differ within each PCN?

***EMR Transferability***

- How will OSCAR EMR centralization/interoperability and scheduling be affected in each PCN after transition?

***Community Pharmacy Engagement***

- How will the Program transition impact community pharmacy engagement and ongoing training and support of shared care of mutual patients?
- How will the scaling of the Program impact community pharmacy engagement initiatives as well as support the shared care of mutual patients?
- What additional infrastructural needs would need to be met after Program transition?

**Probing/Closing Questions**

- What are your thoughts on the future pathway for pharmacy training in primary care?
- Where do you feel your role have had the greatest impact on the Program?
- Generally speaking, what do you think are factors which facilitated implementing the Program?
- Overall, what do you think are some barriers to implementing the Program?
- Is there anything we haven’t talked about that you would like to discuss or share with us today?

**T2 Focus Group Topic Guide - Primary Care Clinical Pharmacists (PCCPs)**

**Introductory/Icebreaker**

- We understand you have been working as a PCCP within the Pharmacists in PCN Program, how would compare your experience over time? How did it evolve compared to when you started?

**UBC Central Support**

- What has been the most/least utilized UBC resources to support clinical care in the PCNs?
- Overall, how has your experience been working with the UBC Program team?
- In your opinion, what could UBC have done differently to further support the PCN clinics to ensure integrating of PCCPs and establishing of the Program?

**Assessments and Continuing Training (Quality Care)**

- What have your experiences been with the quality care assessment aspect of the Program? How does it compare with the first two years?
- What have your experiences been with the professional development aspects of the Program? How does it compare with the first two years?

**PCN-Related Factors**

- What were the pre-existing factors at your PCN which supported your role?
- What were the PCN-related barriers which challenged your ability to practice to your full scope? How did the differ throughout the course of the Program?
- Are you currently practicing to full capacity? If not, what are the barriers that are preventing this?

**EMR Coordination and Access**

- How have the issues/barriers faced regarding EMR coordination across the clinics evolved throughout your time in the Program (access and permissions to utilize EMR)?
- If you have PCN EMR access, have you received training for it?  Was the training adequate to support your full functionality of the PCN EMR?
- What changed and what improvements were made?
- How have the challenges of accessing Pharmanet evolved to optimize your role? How has this barrier been addressed since last year?
- How did these impact your work? Provide examples please.
- What were some workarounds you utilized, if any?

**Technical Needs and Lessons Learned**

- How have the technical needs adapted and changed throughout the Program? What are the lessons learned?
- How has it affected the delivery of care?

**Experiences Working with IPT Members**

- What are some differences that you have observed with different types of care provider practices (FPs vs NPs vs other IPT members)? Does it differ compared to when you started the job?
- To what extent do you feel your expertise as a PCCP is valued by other members of the PCN IPT?
- How do you think your model of care affects interaction with IPT members?
- In terms of patient care, what have been some productive aspects of working with IPT members? What are aspects that leave room for improvement?
- Can you share with us your experiences with case conferences? How did it differ throughout the course of the Program? What were some barriers? What were some supporting factors?

**Intraprofessional Collaboration**

- What is the extent and nature of the interaction between you and other pharmacists? What has been the nature and frequency of the information exchange?
- How did intraprofessional collaboration differ over the course of the Program? More/less interactions?
- What role did the patient play in the cross-communication with the community pharmacist?
- How the relationship building over the last year impacted your work?
- To what extent do you feel your expertise is valued by community pharmacists?
- What role did the Program play in coordinating your interactions with the community pharmacist?
- How was your experience been working with transitions of care with hospital pharmacists in the Program?
- What factors have been identified as enablers of collaboration?
- What factors have been identified as barriers to collaboration?

**Referral Volumes and Program Capacity**

- How have the appointment volumes evolved over the course of the Program?
- How beneficial were the measures taken by the Program to optimize the referral volumes?
- What were the specific approaches for increasing patient referrals that had the biggest impact on patient appointments?
- What did work and did not work?
- In your opinion, what are the factors affecting the referral volumes?

**Patient Follow-up**

- What can you tell us about patient attachment in the Program (e.g., follow-up)?
- Can you tell us about some experiences of patients following up with the Program after hospitalization? How are they different from the first two years of the Program?

**Shared Decision Making**

- Overall, how has the Program supported your role in the SDM process?
- How do you think the Program could have better supported your role in the SDM process?
- Tell us about the extent to which you are able to have latitude and influence in clinical decision making that supports your patients, specifically in resolving DTPs?

**Program Transition**

***UBC Supporting Resources***

- What are the critical elements of the Program at the PCN level that should be retained at the time of transition?
- If the Program was to run for 3 years more as we know it now, what do you think that could be done differently to enhance its implementation?
- If you could think of resources that UBC offered to support Program implementation such as HR (MOAs, Site Coordinators and Quality Care team) and technological support, which elements of UBC central support do you think PCNs would need after Program transition?
- What other lessons have you learned from working in the Program so far and what do you think should be considered for its transition?
- For the Program elements you know/expect to be discontinued, could you elaborate on the level of impact you predict on the Program?
- What do you anticipate as potential barriers and issues post-transition?
- What do you think have been the sources of frustrations during the last 6 months of the transition in respect to organization, communications, and governance?
- Do you think the new proposed structure (what is known so far) can be expected to maintain the Program in the future?
- What are your thoughts/perspectives on the HR needs for site coordinators for PCCPs after Program transition?
- We understand the Ministry has made a decision to not continue UBC MOAs in their role post transition. What are your thoughts on that?

***PCN-Related Factors***

- What factors do you think would affect adoption readiness of PCNs?
- How will PCN acceptability affect transferability of the Program?
- How do you think scalability will differ within each PCN?

**Probing/Closing Questions**

- What are some factors that have an impact on your satisfaction with your current role as a PCCP?
- We understand that you have been advocating for your role as a PCCP within the PCNs, what can tell us about this? How does it compare to the first two years of the Program?
- What are your thoughts on the future pathway for pharmacy training in primary care?
- What are the advantages of central Program delivery by UBC?
- What are the disadvantages of central Program delivery by UBC?
- What do you think could have been done to further support your role within the Program?
- Based on your experience working within the Program, what surprised you the most about your role or the Program so far?
- Is there anything we haven’t talked about that you would like to discuss or share with us today?

**T2 Interview Topic Guide - PCN Administrators**

**Role and Practice**

- What is your role in the PCN?
- How long have you been working in your current role?
- What was the situation like at your PCN when you started working in your current role?
- What were the pre-existing factors at your PCN which supported Program implementation?
- What can you tell us about your experience working with the health authority? How does it support your role in the PCN? (Decision making)

**Experience Working within the PCN**

- What are some enablers for PCN implementation at the Ministry, community, HA, divisions or PCN level?
- What are some barriers for PCN implementation at the Ministry, community, HA, divisions or PCN level?
- What can you tell us about how well your PCN is integrated? Feel free to tell us what you consider to be “good integration”.
- What is your PCN currently working on in terms of its own integration?
- Can you share with us how patients are referred to see the first provider (FP/NP) at your PCN?
- What about cross referrals to other IPT members?
- Does your PCN use patient navigation databases like Pathways or Gateway?
- If yes, can you share with us your experience using that navigation database? Tell us about how Pathways has helped or hindered connection with the Program/PCCP.
- Has your PCN been dealing with EMR and legislation changes (e.g. MediTech) and how has that affected working with the Program?

**Experience with Models of Care**

- What model of care did you start with? What model of care do you work with now? How would you compare these experiences?
- Can you share with us your perspectives on why your PCN switched to the current model of care?
- Can you walk us through how the PCCP works with your regional/centralized hubs?
- What are the barriers to implementing a co-location model in your PCN?
- What would be your preferred model of care? Why?

**Experiences with the UBC Program (central support by UBC)**

- What can you tell us about the quality of coordination by the UBC team?
- What has your experience been working with UBC team (MOAs, Site Coordinators and Quality Care team)?
- How has UBC supported integrating PCCPs into your team?
- In your opinion, how can UBC further support your PCN clinics to ensure integrating of PCCPs and establishing of the Program?
- What are your thoughts on and experiences with the formal communication such as reports generated by UBC to help you get informed about Program updates?
- What are your thoughts on and experiences with other communication materials (emails, newsletter and day to day communication) generated by UBC?
- What kind of challenges have you faced during information sharing from UBC?
- What are your thoughts on and experiences with the IT and technological support provided by UBC for PCCPs? (feedback shared)
- What kind of challenges have you faced regarding technology support from UBC?

**Experiences Working with PCCPs**

- What processes have you had in place to raise awareness about having a PCCP on your IPT and their scope of work?
- What measures have your PCN taken to support patient referrals to a PCCP?
- What are your thoughts on the level of uptake of PCCPs/ patient appointments? Is it going as expected? Why?
- What are some ways that the PCN can do to further help PCCPs integrate into PCNs?
- How does your practice currently work with the PCCPs? Are they physically located in a clinic, or do you connect with them remotely?
- With respect to bringing PCCPs into PCN teams, what is it like having a PCCP on your IPT?
- Do you think that there is a need for PCCPs in this team-based care model?
- What else can you say about working with the PCCPs in your role? How has working with the PCCP affected your workflow?
- Can your share with us your thoughts on the dynamics of team-based care model and how is it functioning in your PCN?
- What can you tell us about the joint educational sessions between primary care providers (FPs/NPs) and PCCPs?
- Does your PCN currently have a Pharmacy Lead or champion that works with the PCCP/Pharmacists in Primary Care Networks Program? Can you share with us your experience working with them and what they do?

**Readiness for Adoption of PCCPs in your PCN**

- Do you think your PCN is ready to work with PCCPs who provide care for complex patients? What can you tell us about that?
- What are your thoughts on what factors may impact the integration of PCCPs among primary care providers?
- What do think is working well with PCCP adoption?
- What about the things that you think are not going well?
- Based on your experience working with the Program, in general, what tends to work well with PCCP introduction?
- What do you think are factors that have enabled and supported the establishment of connections of PCNs with PCCPs?
- What do you think are some challenges or barriers to the establishment of connections of PCNs with PCCPs?
- What is a good way to assess readiness for a PCN to adopt PCCPs into their system or team? (Clinic level/preparedness)
- In your opinion, what could be done to improve integration of PCCPs into primary care?

**Program Transition**

- If you could think of resources that UBC offered to support Program implementation such as HR (MOAs, Site Coordinators and Quality Care team) and technological support, which elements of UBC central support do you think your PCN would need after Program transition?

**Closing/Probing Questions**

- Is there anything we haven’t talked about that you would like to discuss or share with us today?

**T2 Interview Topic Guide - PCN Interprofessional Team (IPT) Members**

**Role and Practice**

- What do you do in the PCN and how long have you worked in this role?
- What has it been like working in this PCN and how does it compare to your previous experiences?
- Do you provide patient care via telehealth? What has that been like?

**Experiences providing care within the PCN**

- What has it been like working in a team-based care setting?
- How is a patient referred to see you?
- How do you refer patients to other IPT members? Is it different from referring to a PCCP?
- Does your PCN use patient navigation databases like Pathways or Gateway? What has that been like?
- What can you tell us about how well your PCN is integrated? Feel free to tell us what you consider to be “good integration”.

**Experiences with The UBC Program Team**

- Do you work with any UBC Program team (i.e. site coordinators?)
- What can you tell us about the quality of coordination by the UBC team?

**Experiences Working with PCCPs**

- What is the perceived need for PCCPs in primary care/PCNs?
- How did you learn about having a PCCP as part of your IPT?
- Do you think that the PCCP’s role/existence in your IPT is widely promoted at the PCN level?
- What is it like having a PCCP on your IPT?
- What are your perceived benefits on the role PCCPs play in caring for patients in PCNs?
- Tell us about an experience you have had working with a PCCP
- Have you conducted any case conferences with a PCCP? What can you tell us about it?
- How has involving a PCCP affected your practice? Has it decreased the amount of time you need to spend on complex patients?
- What do you think are factors that have enabled and supported you when working with a PCCP?
- On the other hand, what kind of challenges have you faced when working with a PCCP?
- Do you think that there is a need for PCCPs in this team-based care model?
- What else can you say about working with the PCCPs in your role?
- What activities performed by a PCCP that you think have resulted in efficiencies of patient care and/or increased your satisfaction?
- Can you share with us your definition of “quality of care” in terms of the PCCP in your IPT?
- In what ways do you feel PCCPs have contributed to providing quality patient care in your IPT?

**Referral Volumes**

- What are the factors affecting the referral volumes to PCCPs?  Model of care?
- What do you think could be done to optimize referrals to PCCPs?

**Closing/Probing Questions**

- Is there anything we haven’t talked about that you would like to discuss or share with us today?

**T2 Interview Topic Guide – Family Physicians/Nurse Practitioners**

**Experiences Working with PCCPs**

- How did you learn about having a PCCP as part of your IPT?
- Do you think that the PCCP’s role/existence in your IPT is widely promoted at the PCN level?
- How has involving a PCCP affected your practice? Has it decreased the amount of time you need to spend on complex patients?
- What do you think are factors that have enabled and supported you when working with a PCCP?
- On the other hand, what kind of challenges have you faced when working with a PCCP?
- What activities performed by a PCCP that you think have resulted in efficiencies of patient care and/or increased your satisfaction?
- What are your perceived benefits on the role PCCPs play in caring for patients in a team-based care model? What is the perceived need for PCCPs in primary care/PCNs?
- Can you share with us your definition of “quality of care” in terms of the PCCP in your IPT? And in what ways do you feel PCCPs have contributed to providing quality patient care in your IPT?
- How did you work together with the PCCP to discuss and implement their recommendations following a patient appointment? What could have been done differently? What worked? What didn’t?
- What else can you say about working with the PCCPs in your role?

**Closing/Probing Questions**

- Is there anything we haven’t talked about that you would like to discuss or share with us today?
